# Estimation of the Relative Severity of Floods in Small Ungauged Catchments for Preliminary Observations on Flash Flood Preparedness: A Case Study in Korea

**DOI:** 10.3390/ijerph9041507

**Published:** 2012-04-18

**Authors:** Eung Seok Kim, Hyun Il Choi

**Affiliations:** 1 Department of Civil Engineering, Sunmoon University, 100, Kalsan-ri, Tangjeong-myeon, Asan-si, Chungnam-do 336-708, Korea; Email: hydrokes@sunmoon.ac.kr; 2 Department of Civil Engineering, Yeungnam University, 214-1, Dae-dong, Gyeongsan-si, Gyeongbuk-do 712-749, Korea

**Keywords:** flash flood index, flash flood preparedness, flood severity, runoff hydrograph

## Abstract

An increase in the occurrence of sudden local flooding of great volume and short duration has caused significant danger and loss of life and property in Korea as well as many other parts of the World. Since such floods usually accompanied by rapid runoff and debris flow rise quite quickly with little or no advance warning to prevent flood damage, this study presents a new flash flood indexing methodology to promptly provide preliminary observations regarding emergency preparedness and response to flash flood disasters in small ungauged catchments. Flood runoff hydrographs are generated from a rainfall-runoff model for the annual maximum rainfall series of long-term observed data in the two selected small ungauged catchments. The relative flood severity factors quantifying characteristics of flood runoff hydrographs are standardized by the highest recorded maximum value, and then averaged to obtain the flash flood index only for flash flood events in each study catchment. It is expected that the regression equations between the proposed flash flood index and rainfall characteristics can provide the basis database of the preliminary information for forecasting the local flood severity in order to facilitate flash flood preparedness in small ungauged catchments.

## 1. Introduction

Climate change poses one of the greatest threats to the World’s environment, already contributing to visible impacts on human health, economic activity, food security, agriculture, natural environment and ecosystems, water and other natural resources, physical infrastructure, and so on. The Fourth Report of the Intergovernmental Panel on Climate Change (IPCC) has brought to the fore the severity and global dimension of the impacts of climate change. It was reported that while the number of rainy days has decreased, the occurrence of more intense rainfall events has increased in most parts of Asia, which has led to severe floods, landslides, and debris and mud flows [1]. The climate change could be even more noticeable in Korea. According to the Korea Meteorological Administration (KMA), the Korean Peninsula is warming at more than twice the speed of the global average, and the yearly precipitation has increased approximately 19 percent in the period of 1912–2008 with an increase in summer precipitation and localized torrential downpours. As the temporal and spatial fluctuations of the precipitation are expected to increase, most watersheds in the Korean Peninsula have climatic and geomorphic vulnerability to flash floods caused by localized convective storms of short duration over small catchments. Flash flooding is a sudden local flood of great volume and short duration, which is now a common natural disaster in Korea. The annual natural disaster bulletin of Korea National Emergency Management Agency (KNEMA) reported that some watershed areas were inundated with the rapid debris flow runoff raising some flood damage such as bank erosion and bridge collapse [2]. It is, however, quite tough to cope with such types of flood damage, because local flooding in small watersheds rises quite quickly with little or no advance flood warning. There is a limit on predicting a sudden local flood in small watersheds through most present flood forecasting systems based on a rainfall-runoff model with high computational time [3,4,5,6]. It is therefore required to characterize the hydrologic behavior of local flooding that occurs in small catchments with the short flood response time in order to establish an appropriate and effective emergency preparedness and response system to flash flood disasters.

Many studies have examined flash floods mainly from a climatological perspective, especially focused on the temporal and spatial characteristics of rainfall, such as for the determination of climatological characteristics of flash floods and extreme rain events [7,8,9,10,11], and recently for the use of radar information in flash flood prediction [12,13,14,15,16]. From the hydrological perspective on flash floods, post-flood estimations of peak discharges have been extensively conducted in various countries to provide the effective documentation of flash flood events and enhance understanding on regional behavior of extreme events [17,18,19,20,21,22,23,24,25,26]. In order to better understand the hydro-meteorological processes leading to flash floods, the EU Hydrometeorological Data Resources and Technology for Effective Flash Flood Forecasting Project (HYDRATE) was established, and has studied on enhancing the capability of flash flood forecasting [27,28,29,30,31]. In the US, for the flash flood guidance (FFG)-based flash flood warnings and watches issued by National Weather Service (NWS), many studies have focused on derivation of the geomorphological and climatological instantaneous unit hydrograph, estimation of the threshold runoff, development of geographic information system (GIS)-based procedures in support of the FFG, and so on [3,32,33,34,35]. On the other hand, Kyiamah [36] and Bhaskar *et al.* [37] initially characterized flash floods from a runoff perspective using flood runoff hydrographs. They presented a flash flood index evaluated by relative severity factors quantifying characteristics of the observed hydrographs such as rising curve gradient, flood magnitude ratio, and flood response time in order to distinguish flash floods from other floods. Following the methodology suggested by Bhaskar *et al.* [37], Jung [38] estimated the flash flood index for several flood events of the Bo-chung River basin in Korea. However, these studies have the problematic issue of quantifying the three relative severity factors by each using a different ordinal scale of assignment where the choice of class intervals is to some extent arbitrary. Kim and Kim [5] estimated the flash flood index for investigating the relative severity of flash floods in the Han River basin with 101 flood events, and quantified the flash flood severity for some flood events caused by heavy rainfall in July of 2006. Whereas most previous studies computed the flash flood index directly from the observed flood hydrographs, Kim and Choi [39] made an attempt to evaluate vulnerability to extreme flash floods in design storms through the flash flood index for small ungauged catchments where rainfall observations are usually available.

This study has modified the flash flood index from Bhaskar *et al.* [37], and presented a new methodology to measure the relative severity of local flash flooding in small ungauged catchments by a dimensionless flash flood index from the normalized relative severity factors on the same scale ratio to the highest recorded maximum value. This flash flood index is obtained by quantifying the characteristics of hydrographs generated from a rainfall-runoff model, Hydrologic Engineering Center-Hydrologic Modeling System (HEC-HMS), for the annual maximum rainfall series of long-term observed data. The developed flash flood index is implemented in the two selected small ungauged basins of Korea, the Oui-mi River basin located in the hilly region and the Mae-gok River basin in the relatively flat region. The regression analysis of relationships between the flash flood index and rainfall characteristics presented in this study can provide better understanding of the hydrologic behavior of local flash flooding for the preliminary information on an emergency preparedness and response system to flash flood disasters in small ungauged catchments.

## 2. Study Catchments

**Figure 1:**
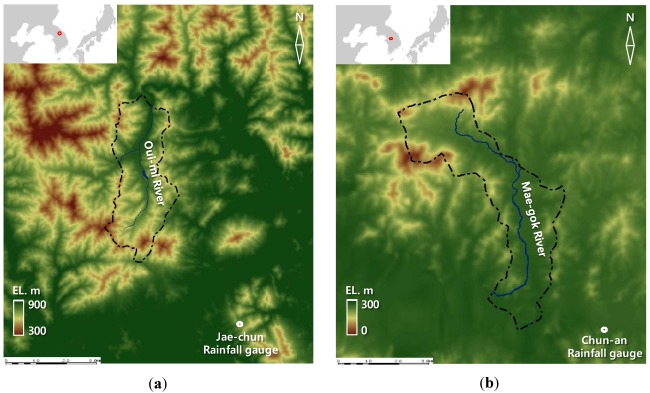
Basin maps for (**a**) the Oui-mi River and (**b**) the Mae-gok River.

This study selected the two small ungauged natural catchments, the Oui-mi River basin in a hilly slope terrain and the Mae-gok River basin with a flat topography, for study basins around which rainfall gauge stations have recorded the long-term hourly rainfall data. The location and shape of study basins are shown in Figure 1, and the summary of the characteristics of the two basins under study are presented in Table 1.

**Table 1:** The summary of characteristics for the two study basins.

Basin Name	Bain Area A (km^2^)	Basin Length L (km)	Basin Width A/L (km)	Shape Factor A/L^2^	Average Elevation (EL.m)	Average Slope (%)
The Oui-mi River	16.74	7.52	2.23	0.30	544.9	53.4
The Mae-gok River	35.48	11.25	3.15	0.28	65.0	9.6

The Oui-mi River basin is a small hilly drainage basin of 16.74 km^2^ size and 7.52 km length, located between 128°11′~128°12′E and 37°15′~37°16′N [40]. The basin is a natural drainage area where land cover types consist mainly of forests (86.3%) and croplands (11.2%). This basin located in a mountainous inland area is characterized by a continental climate with cold winters and hot summers. The average annual temperature is 10.3 °C, and the average monthly temperature ranges from −2.7 °C in January to 23.4 °C in August. Annual maximum rainfall data was collected for the period of 1973–2008 from the Jae-chun gauge station which has a self-recording rain gauge managed by the Korea Meteorological Administration. The data showed that the annual mean rainfall volume is 1,322.5 mm, greater than the national average of 1,283.0 mm over the same period, as a result of proximity to the mountainous region. The highest recorded maximum depth of a rainfall event was 228.5 mm in 11 September 1990. 

The Mae-gok River basin was also selected to investigate flood characteristics for the small flat drainage area and compare results with those in the Oui-mi River basin. This river basin is located between 127°02′~127°08′E and 36°47′~36°52′N with 35.48 km^2^ size and 11.25 km length [41]. Land cover types within the basin are mainly 44% croplands and 36% forests. Annual maximum rainfall event series during 1973–2008 was collected from a self-recording rain gauge in the Chun-an gauge station managed by the Korea Meteorological Administration. The annual mean rainfall volume is 1,235.9 mm, little less than the national average rainfall over the same period, and the highest recorded maximum depth of a rainfall event was 262.5 mm in 9 August 1995. The average annual temperature is 11.6 °C, and the average monthly temperature ranges from −3.0 °C in January to 24.9 °C in August.

## 3. Estimation of Flash Flood Severity

This study presents a new flash flood index estimated from the simulated runoff hydrographs for the annual maximum precipitation series to quantify the severity of flash floods in small ungauged catchments. Bhaskar *et al.* [37] characterized the flash flood severity using a flash flood indexevaluated directly from the observed hydrograph characteristics such as the rising curve gradient *K*, the flood magnitude ratio *M*, and the flood response time *T*. These characteristics were quantified as the relative severity factors *RK*, *RM*, and *RT*, respectively, by using each different ordinal scale of assignment where the choice of class intervals is to somewhat arbitrary. Since Bhaskar *et al.* [37] presented the flash flood index *RF* by the sum of such problematic severity factors on different ordinal scale values, Kim and Choi [39] computed all relative severity factors at the same scale ratio to each highest recorded maximum value. They also demonstrated that the flash flood index from the two relative severity factors *RK* and *RM*, which avoids double-counted relative severity factors with similar characteristics such as *RK* and *RT*, is more adequate to estimate the relative flood severity in small ungauged catchment. This study determines the flash flood index *FI* by the average of the two relative severity factors *RK *and *RM* standardized by the highest maximum value for flash flood events only. Details of the flash flood indexing procedure are discussed below.

### 3.1. Flood Runoff Hydrographs

Flood runoff hydrographs are generated from a rainfall-runoff model, HEC-HMS [42], using annual maximum precipitation series for past 36 years during 1973–2008 from the Jae-chun gauge station for the Oui-mi River basin and the Chun-an gauge station for the Mae-gok River basin, respectively. The annual maximum precipitation series for the two rain gauge stations were constituted by selecting the maximum depth for a rainfall period among all rainfall events recorded in each year. The NRCS (Natural Resources Conservation Service) curve number method [43] is used for the loss rate method, and the Clark unit hydrograph [44] is employed for the transform method for rainfall-runoff simulations in both basins. The NRCS curve number is averaged for the Oui-mi River basin as 70.10 and for the Mae-gok River basin as 87.92, respectively. The storage coefficient of the Clark unit hydrograph is estimated for the Oui-mi River basin as 1.18 h and for the Mae-gok River basin as 2.02 h, respectively. Note that both parameter values are directly referred in the basic plan reports for the river maintenance works by Wonju city [40] for the Oui-mi River basin, and Chungcheongnam province [41] for the Mae-gok River basin, respectively, which were used for estimating the design flood discharges that have been officially announced for both study basins. The maximum peak flood discharge of 183.3 m^3^/s is estimated for the Oui-mi River basin in 5 August 2007 (see the second column of Table 2), and the maximum peak flood discharge of 352.8 m^3^/s is computed for the Mae-gok River basin in 9 August 1995 (see the second column of Table 3), respectively, among the annual maximum simulated hydrographs during the analysis period of 1973–2008.

**Table 2:** Summary of runoff and indexing characteristics along with rainfall data for the selected flood events among 36-year annual maximum series in the Oui-mi River basin.

No	Flood Runoff Characteristics				Flash Flood Indexing Parameters					Rainfall Characteristics								
	Flood Event Date (mm/dd/yy) [1]	Flood Peak Discharge Q_p _(m^3^/s) [2]	Time to Flooding Start T_b _(h) [3]	Time to Flooding Peak T_p _(h) [4]	Flood Magnitude Ratio M [5]	Rising Curve Gradient K (mm/h^2^) [6]	Relative Flood Severity RM [7]	Relative Flood Severity RK [8]	Flash Flood Index FI (%) [9]	Average Rainfall Intensity I_a _(mm) [10]	Max 1-h Rainfall R_1h _(mm) [11]	Max2-h Rainfall R_2h _(mm) [12]	Max3-h Rainfall R_3h _(mm) [13]	Max4-h Rainfall R_4h _(mm) [14]	Max5-h Rainfall R_5h _(mm) [15]	Max6-h Rainfall R_6h _(mm) [16]	Total Rainfall Depth R_t _(mm) [17]	Rainfall Duration Time D (h) [18]
1	07/22/80	71.7	16.9	17.0	1.1	11.7	0.4	0.6	50.8	6.6	43.0	53.2	75.4	85.6	91.1	95.9	132.6	20.0
2	07/19/86	80.5	6.5	7.0	1.2	6.9	0.4	0.4	40.4	6.4	32.0	58.0	69.0	78.5	86.0	97.0	134.2	21.0
3	07/22/87	70.0	3.8	4.0	1.1	4.4	0.4	0.2	30.9	8.2	41.5	57.5	67.5	82.0	92.0	101.5	187.5	23.0
4	07/14/88	111.5	8.6	12.0	1.7	3.0	0.6	0.2	38.3	14.0	33.0	57.0	75.5	99.5	118.0	134.0	223.5	16.0
5	07/26/89	77.4	5.8	7.0	1.2	2.2	0.4	0.1	27.0	6.2	34.0	67.5	85.5	89.5	95.0	99.0	143.0	23.0
6	09/11/90	92.8	23.3	24.0	1.4	8.3	0.5	0.4	47.5	9.5	38.5	72.0	88.0	93.5	94.5	102.0	228.5	24.0
7	07/13/93	70.4	12.8	13.0	1.1	7.5	0.4	0.4	39.1	7.5	30.5	42.0	52.5	63.5	69.0	75.0	158.5	21.0
8	06/30/94	123.6	18.9	20.0	1.9	11.8	0.7	0.6	65.1	8.5	37.0	68.5	90.5	94.0	100.0	102.5	196.5	23.0
9	07/01/97	98.7	16.6	18.0	1.5	5.1	0.5	0.3	40.4	7.2	49.5	56.5	63.5	69.5	73.5	79.0	166.5	23.0
10	06/30/01	98.3	4.1	5.0	1.5	8.4	0.5	0.4	49.2	17.8	41.0	72.0	87.0	93.5	104.0	106.5	106.5	6.0
11	07/16/06	67.5	14.5	15.0	1.0	1.2	0.4	0.1	21.5	8.5	22.5	42.0	54.5	71.0	86.0	91.0	203.0	24.0
12	08/05/07	183.3	5.6	7.0	2.8	18.8	1.0	1.0	100.0	18.7	68.0	122.5	149.0	161.0	171.5	180.5	186.5	10.0
13	07/24/08	70.6	17.9	19.0	1.1	1.1	0.4	0.1	22.3	4.0	49.0	63.0	68.0	69.5	74.0	77.5	96.5	24.0
Maximum		183.3	23.3	24.0	2.8	18.8	1.0	1.0	100.0	18.7	68.0	122.5	149.0	161.0	171.5	180.5	228.5	24.0
Minimum		67.5	3.8	4.0	1.0	1.1	0.4	0.1	21.5	4.0	22.5	42.0	52.5	63.5	69.0	75.0	96.5	6.0

**Table 3:** Summary of runoff and indexing characteristics along with rainfall data for the selected flood events among 36-year annual maximum series in the Mae-gok River basin.

No	Flood Runoff Characteristics				Flash Flood Indexing Parameters					Rainfall Characteristics								
	Flood Event Date (mm/dd/yy) [1]	Flood Peak Discharge Q^p^ (m^3^/s) [2]	Time toFlooding StartT_b_ (h) [3]	Time toFlooding PeakT_p_ (h) [4]	FloodMagnitudeRatioM [5]	RisingCurveGradientK (mm/h^2^) [6]	RelativeFloodSeverityRM [7]	RelativeFloodSeverityRK [8]	FlashFloodIndexFI (%) [9]	AverageRainfall IntensityI_a_ (mm) [10]	Max1-h RainfallR_1h_ (mm) [11]	Max2-h RainfallR_2h_ (mm) [12]	Max3-h RainfallR_3h_ (mm) [13]	Max4-h RainfallR_4h_ (mm) [14]	Max5-h RainfallR_5h_ (mm) [15]	Max6-h RainfallR_6h_ (mm) [16]	TotalRainfall DepthR_t_ (mm) [17]	RainfallDuration TimeD (h) [18]
1	08/14/76	209.2	1.8	3.0	1.4	5.4	0.6	0.6	57.2	10.5	49.5	94.5	107.5	115.4	115.4	115.4	125.6	12.0
2	09/06/77	180.7	6.9	8.0	1.2	3.0	0.5	0.3	40.7	7.4	37.0	73.5	84.5	91.0	100.6	104.6	147.7	20.0
3	08/16/78	189.2	7.9	9.0	1.3	3.9	0.5	0.4	46.5	13.7	27.5	54.0	76.0	101.5	117.0	119.0	123.0	9.0
4	07/14/80	198.5	4.2	5.0	1.3	6.8	0.6	0.7	62.7	15.5	46.0	73.0	96.5	104.5	107.5	108.0	108.5	7.0
5	07/28/82	180.7	7.0	8.0	1.2	3.6	0.5	0.4	43.6	8.3	44.5	61.0	86.5	95.0	104.0	116.0	166.0	20.0
6	07/04/84	210.6	6.6	8.0	1.4	4.5	0.6	0.5	52.5	7.5	39.5	58.0	75.0	91.0	114.5	133.0	158.0	21.0
7	07/19/86	171.3	4.3	5.0	1.2	3.3	0.5	0.3	41.2	13.4	34.0	57.0	83.0	106.0	111.0	114.0	120.2	9.0
8	07/21/87	175.0	17.1	18.0	1.2	3.3	0.5	0.3	41.6	7.8	31.5	53.5	79.5	85.5	88.5	89.0	149.0	19.0
9	08/27/92	179.1	9.7	11.0	1.2	2.5	0.5	0.3	38.0	7.6	29.5	49.5	61.0	90.5	107.5	119.5	159.5	21.0
10	08/09/95	352.8	3.9	6.0	2.4	9.9	1.0	1.0	100.0	23.9	67.5	103.5	132.5	156.5	175.5	200.5	262.5	11.0
11	07/01/97	209.7	11.8	13.0	1.4	5.5	0.6	0.6	57.4	8.0	33.0	62.5	86.0	99.5	108.5	113.5	151.5	19.0
12	08/07/01	200.5	5.6	7.0	1.4	3.8	0.6	0.4	47.6	18.4	35.5	65.5	80.5	91.0	113.0	128.0	128.5	7.0
13	08/07/02	240.1	8.6	13.0	1.6	2.2	0.7	0.2	44.9	14.9	37.5	64.0	86.0	105.0	133.0	161.0	239.0	16.0
14	09/18/05	164.7	9.0	10.0	1.1	1.8	0.5	0.2	32.3	10.2	33.5	56.5	81.0	89.0	99.5	103.0	112.0	11.0
Maximum		352.8	17.1	18.0	2.4	9.9	1.0	1.0	100.0	23.9	67.5	103.5	132.5	156.5	175.5	200.5	262.5	21.0
Minimum		164.7	1.8	3.0	1.1	1.8	0.5	0.2	32.3	7.4	27.5	49.5	61.0	85.5	88.5	89.0	112.0	7.0

### 3.2. Flood Magnitude Ratio

Bhaskar *et al.* [37] presented the flood magnitude ratio *M*, which means a ratio of the peak flood discharge *Q_p_* to the long-term average discharge *Q_a_*:


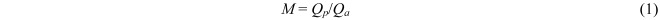


The long-term average discharge *Q_a_* is also not available in ungauged catchments. Furthermore, we need to define a threshold discharge distinguishing a flash flood event with the potential flooding risk from other normal stormwater runoff events for a watershed. For flash flood preparedness guidance purposes, a threshold runoff is defined as a ratio of stream flow at the bankfull stage to the unit hydrograph peak flow for a watershed [32]. The bankfull discharge can be determined by identifying the bankfull stage and then determining the discharge associated with the bankfull stage. Although many field indicators have been proposed, defining the bankfull stage is complicated [45]. Due to difficulties in the identification of bankfull discharge and the lack of measurements for the two ungauged study catchments, this study assumes the 2-year return period discharge as a threshold discharge following Wolman and Leopold [46]. Hence, the bankfull discharge *Q_b_* is replaced for *Q_a_* in Equation (1) to represent the relative amount of flood to the flooding threshold discharge:


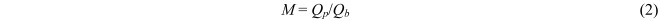


The estimated threshold discharges for the 2-year return period are 64.9 and 147.1 m^3^/s in the Oui-mi River basin and the Mae-gok River basin, respectively. The value of the flood magnitude ratio *M* varies from 1.0 to 2.8 for the Oui-mi River basin as shown in column 5 of Table 2, and varies from 1.1 to 2.4 for the Mae-gok River basin as shown in column 5 of Table 3, respectively. The flash flooding situation can be characterized only for flood events with the flood magnitude ratio *M* greater than 1.

### 3.3. Rising Curve Gradient

Since floods that have short lag times between the storm and the peak discharge result in fast runoff concentration and in consequences high flooding hazard, a flash flood characterized by a rapid rate of rise with a high velocity means that the event is predicted to be hazardous. Hence, the rising curve gradient of hydrographs can represent the typical characteristics of flash floods that happen quickly at some location over a short time period. Bhaskar *et al.* [37] described the rising limb of hydrographs as an exponential equation, and thus the rising curve gradient *K* (/day) can be computed as:


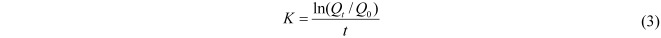


where *Q_0_* is the specified initial discharge, and *Q_t_* is the discharge at a later time *t* close to the time of peak. This exponential function popularly used for hydrograph recession curves has a difficulty in defining the specified initial discharge *Q_0_* for the simulated runoff hydrographs, which is usually zero. Also, sometimes finding the starting time of flood runoff hydrographs can be arbitrary and ambiguous in accordance with the shape of the rising limb. Moreover, both study basins have occasionally recorded a certain rainfall pattern that the incipient rain is moderate but torrential rain occurs abruptly in the middle of a rainfall duration time. Hence, this study expresses the rising limb gradient of simulated hydrographs using a mean slope gradient approximation between occurrence times *T_p_* and *T_b_* corresponding to the peak discharge *Q_p_* and the bankfull discharge *Q_b_* in the rising limb of a hydrograph, respectively:


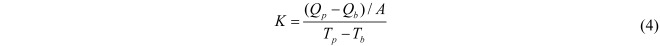


where *A* is the drainage area. The rising curve gradient *K* is computed for the specific discharge (discharge per unit area) with a unit of mm/h^2^. The range of values for the rising curve gradient *K* is from 1.1 to 18.8 mm/h^2^ for the Oui-mi River basin as shown in column 6 of Table 2, and is from 1.8 to 9.9 mm/h^2 ^for the Mae-gok River basin as shown in column 6 of Table 3, respectively. Since the rising curve gradient represents the steepness of the rising limb of flood hydrographs, the large values of parameter *K *can be associated with a rapid local flood of great volume.

### 3.4. Flash Flood Index

The characteristics of flood hydrographs need to be integrated for an overall value to evaluate flash flood severity. Because the rising curve gradient *K* and the flood magnitude ratio *M* are measured at different scales and units, they need to be transformed into a common domain prior to combining them. In order to contribute equally to the analysis, the flood hydrograph characteristics are standardized by the maximum value:


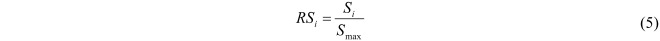


where *S_i_* is the evaluation score for the flood hydrograph characteristics such as *K* and *M* for the *i*th flood event, *S_max_* is the highest value of *S_i_*, and *RS_i_* is the relative severity factor of *S_i_*. The flash flood index *FI* for each flood event is computed by taking the average of the two relative severity factors such as *RK* and *RM*:


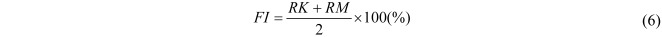


Note that only when the flood magnitude ratio *M* is greater than 1, *i.e.*, the peak discharge of a flood event is greater than the threshold bankfull discharge, the flash flood index is computed to distinguish a flash flood event from other normal stormwater runoff events. Consequently among the 36 annual maximum simulated hydrographs in the analysis period of 1973–2008 for the two study catchments, 13 and 14 floods are selected for flash flooding events in the Oui-mi River basin and the Mae-gok River basin as summarized in Table 2 and Table 3, respectively.

## 4. Flash Flood Preparedness Information

**Figure 2:**
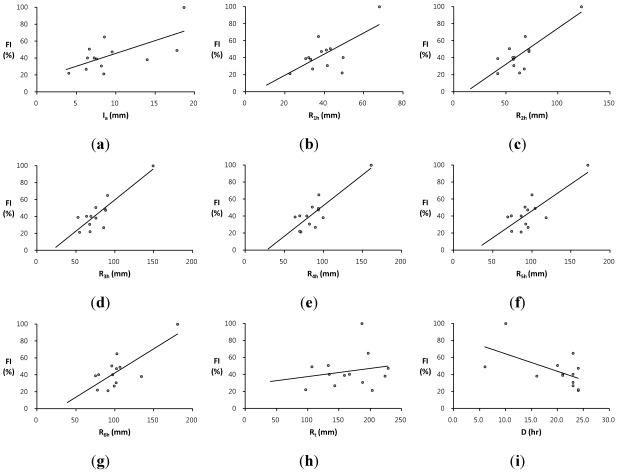
The comparison of trends between the flash flood index *FI *and rainfall characteristics such as; (**a**) the average rainfall intensity *I_a_*; (**b**) the 1-h maximum rainfall depth *R_1h_*; (**c**) the 2-h maximum rainfall depth *R_2h_*; (**d**) the 3-h maximum rainfall depth *R_3h_*; (**e**) the 4-h maximum rainfall depth *R_4h_*; (**f**) the 5-h maximum rainfall depth *R_5h_*; (**g**) the 6-h maximum rainfall depth *R_6h_*; (**h**) the total rainfall depth *R_t_*; and (**i**) the rainfall duration time *D* in the Oui-mi River basin.

**Figure 3:**
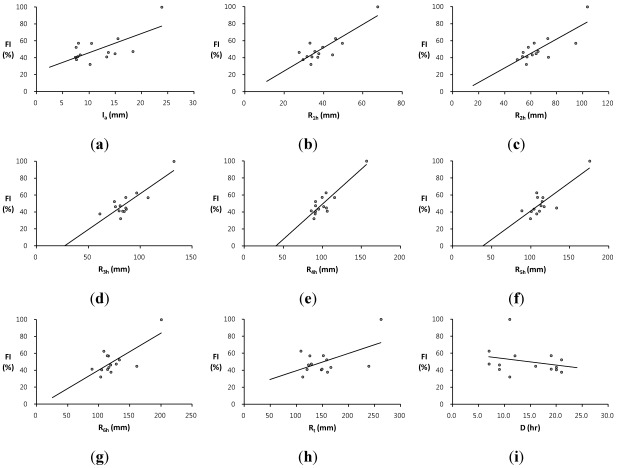
The comparison of trends between the flash flood index *FI *and rainfall characteristics such as; (**a**) the average rainfall intensity *I_a_*; (**b**) the 1-h maximum rainfall depth *R_1h_*; (**c**) the 2-h maximum rainfall depth *R_2h_*; (**d**) the 3-h maximum rainfall depth *R_3h_*; (**e**) the 4-h maximum rainfall depth *R_4h_*; (**f**) the 5-h maximum rainfall depth *R_5h_*; (**g**) the 6-h maximum rainfall depth *R_6h_*; (**h**) the total rainfall depth *R_t_*; and (**i**) the rainfall duration time *D* in the Mae-gok River basin.

Since local flooding in small watersheds rises quite quickly with little or no advance warning to prevent flash flood damage, flash flood forecasting does not require a complex numerical model consuming high computational time. Therefore, the analysis of the relationship between rainfall and runoff characteristics is important for understanding and forecasting flash flooding to promptly provide the preliminary observations on the effective emergency preparedness and initial response information to local flooding with a short period of time over a small area. This study has examined the relationship between the proposed flash flood index *FI* and rainfall characteristics for the selected flood events among 36-year annual maximum series in the two study basins. The analysis is performed by creating scatter plots and regression equations between the flash flood index *FI* and rainfall data such as the average rainfall intensity *I_a_*, the maximum rainfall depths for the 1-h, 2-h, 3-h, 4-h, 5-h, and 6-h durations, *R_1h_*, *R_2h_*, *R_3h_*, *R_4h_*, *R_5h_*, and *R_6h_*, respectively, the total rainfall depth *R_t_*, and the rainfall duration time *D*. The average rainfall intensity means the total amount of rainfall for a storm event divided by the duration of the storm. Table 2 and Table 3 present flash flood index *FI* along with rainfall characteristics and runoff results for the two study basins, the Oui-mi River basin and the Mae-gok River basin, respectively. The scatter plots of the flash flood index *FI versus* each rainfall data are illustrated in Figure 2 for the Oui-mi River basin and Figure 3 for the Mae-gok River basin, respectively. Table 4 summarizes and compares the regression equations of the flash flood index *FI* corresponding to each rainfall data in the two study basins.

**Table 4:** Regression equations for relations between the flash flood index *FI* and rainfall characteristics.

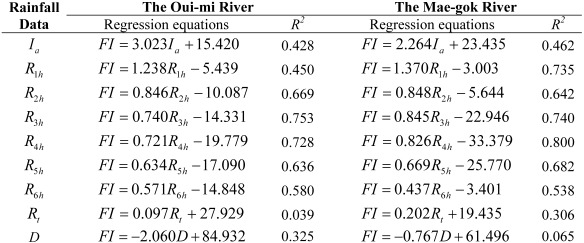

The flash flood index *FI* shows the strong relation to some rainfall data with relatively high coefficients of determination *R^2^* for each study basin. It demonstrates that the flash flood index *FI* can be used as a key indicator to estimate the relative flood severity in these study basins. The Oui-mi River basin has the highest relation between the flash flood index *FI* and the 3-h maximum rainfall depth *R_3h_* with the coefficient of determination *R^2^* of 0.753 as shown in Figure 2d. The trend between the flash flood index *FI* and the 4-h maximum rainfall depth *R_4h_* shows the best-fit line with the coefficient of determination of 0.800 for the Mae-gok River basin as illustrated in Figure 3e. The flash flood index *FI* shows the highest linear relation with the rainfall for a shorter duration in the Oui-mi River basin located at the mountainous region with a smaller area size than in a relatively larger flat watershed of the Mae-gok River basin. It is also observed that the flash flood index *FI* has weak and limited relationship to the average rainfall intensity *I_a_* as well as the total rainfall amount *R_t_* and duration *D* in both study basins as shown in Figure 2 and Figure 3(a,h,i). These results support that a local flash flood in small watersheds is mainly caused by heavy or excessive rainfall in a short period of time. Overall, the coefficients of determination *R^2^* for regression equations in the Oui-mi River basin are less than those in the Mae-gok River basin as summarized in Table 4. 

## 5. Discussion and Conclusions

This study has characterized the severity of flash floods using a dimensionless index describing characteristics of hydrographs generated from a rainfall-runoff model for the long-term observed rainfall data in small ungauged catchments. A new flash flood index *FI* is determined by the average of relative severity factors *RK* for the rising curve gradient *K* and *RM* for the flood magnitude ratio *M*, which are standardized by the highest recorded maximum value. The developed new flash flood index *FI* was implemented for the two selected small ungauged basins in Korea, the Oui-mi River basin for the hill slope terrain region and the Mae-gok River basin for the flat region. Since most current flood forecasting and warning systems based on a rainfall-runoff model are not adequate for use in predicting local flooding that occurs in small catchments with the short flood response time, it is necessary to predict the flash flood severity directly by rainfall patterns for the effective emergency preparedness and initial response information to flash flooding. This study has therefore investigated the relationship of rainfall characteristics to the flash flood index *FI* estimated in the two study basins.A stronger relation of the flash flood index *FI* to the short term rainfall rather than the total rainfall depth illustrates that heavy or excessive rainfall in a short period of time mainly causes local flash flooding in small watersheds. The proposed flash flood index *FI* that has a very high relation to linear of a certain rainfall pattern can measure the relative flood severity of a flood event to the highest recorded maximum flood level, which can promptly provide the preliminary information for use in the flash flood preparedness. In comparison of results from the two study basins, it is observed that the flood behavior of the Oui-mi River basin located at the mountainous region with the smaller area size is strongly influenced by the excessive rainfall in a shorter period of time as compared with the result from the Mae-gok River basin, a relatively larger flat watershed. The coefficients of determination *R^2^* in the Mae-gok River basin are much higher than those in the Oui-mi River basin for most regression equations. It is partially implicated in the use of point rainfall data measured around the basin, which may not adequately capture high spatial variation of rainfall over the hilly terrain region of the Oui-mi River basin, while this effect is less in the flat region of the Mae-gok River basin. It is therefore expected that availability of higher spatial resolution rainfall data may provide a significant improvement to flash flood forecasting in order to cope with the consistent treat of flash flood disasters. Note that this proposed flash flood index does not present any threshold indicators such as the flash flood guidance that should be associated with the current soil moisture condition. For use of the flash flood index *FI* in a practical flash flood warning or alert system in the future, more tests and implementations need to be conducted with the real severity and damages reported from past floods in a large number of watersheds.

The key to flash flood forecasting is to quickly predict when the flood is above a predetermined threshold level, where a bankfull discharge can occur. The further research needs to investigate the uncertainty in determining of a threshold flooding discharge assumed as the 2-year return period flow in this study. Because the recurrence interval relations for the bankfull discharge are intrinsically different for channels, field verification is recommended to insure that the selected discharge can reflect the bankfull stage on the channel. Alternatively, regionalization analysis of several values from a few gauged catchments can be used to estimate the bankfull discharge for ungauged catchments. The flash flood index *FI* is based on flood runoff hydrographs simulated from a rainfall-runoff model, HEC-HMS. Since the HEC-HMS model simulated all time series of annual maximum floods systematically for this analysis no matter how much the simulation results depart from the actual runoff, it can be reasonable for the flash flood index *FI* from the simulated hydrographs to measure the relative severity of a flood event to the highest maximum recorded flood. The uncertainty of the HEC-HMS model itself, albeit it is a generalized modeling system designed to simulate the rainfall-runoff processes of many various watersheds, and calibration parameters used in the rainfall-runoff simulations could be investigated in another research if necessary. Although the current relation results between the flash flood index *FI* and rainfall characteristics are not conclusive for forecasting local flash floods, it is expected that the proposed flash flood indexing methodology can provide the basic database of preliminary observations for use in an emergency preparedness and response system to flash flood disasters.

## References

[1] Intergovernmental Panel on Climate Change (IPCC) (2007). Fourth Assessment Report, Climate Change 2007—Summary for Policymakers.

[2] Korea National Emergency Management Agency (KNEMA) (2010). The Annual Natural Disaster Bulletin.

[3] Sweeney T.L. (1992). Modernized Areal Flash Flood Guidance; NOAA Technical Report NWS HYDRO 44.

[4] Shin H.S., Kim H.T., Park M.J. (2004). The study of the fitness on calculation of the flood warning trigger rainfall using GIS and GCUH. J. Korea Water Resour. Assoc..

[5] Kim B.S., Kim H.S. (2008). Estimation of the flash flood severity using runoff hydrograph and flash flood index. J. Korea Water Resour. Assoc..

[6] Marchi L., Borga M., Preciso E., Gaume E. (2010). Characterisation of selected extreme flash floods in Europe and implications for flood risk management. J. Hydrol..

[7] Doswell C.A. Flash Flood-Producing Convective Storms. Proceedings of the U.S.-Spain Workshop on Natural Hazards.

[8] Lapenta K.D., McNaught B.J., Capriola S.J., Giordano L.A., Little C.D., Hrebenach S.D., Carter G.M., Valverde M.D., Frey D.S. (1995). The challenge of forecasting heavy rain and flooding throughout the Eastern Region of the National Weather Service. Part I: Characteristics and events. Weather Forecast..

[9] Opitz H.H., Summer S.G., Wert D.A., Snyder W.R., Kane R.J., Brady R.H., Stokols P.M., Kuhl S.C., Carter G.M. (1995). The challenge of forecasting heavy rain and flooding throughout the Eastern Region of the National Weather Service. Part II: Forecast techniques and applications. Weather Forecast..

[10] Smith J.A., Baeck M.L., Zhang Y., Doswell C.A. (2001). Extreme rainfall and flooding from supercell thunderstorms. J. Hydrometeorol..

[11] Rogash J.A., Racy J. (2002). Some meteorological characteristics of significant tornado events occurring in proximity to flash flooding. Weather Forecast..

[12] Borga M., Anagnostou E.T., Frank E. (2000). On the use of real-time radar rainfall estimates for flood prediction in mountainous basins. J. Geophys. Res..

[13] Salek M., Brezkova L., Novak P. (2006). The use of radar in hydrological modeling in the Czech Republic—Case studies of flash floods. Nat. Hazards Earth Syst. Sci..

[14] Vivoni E.R., Entekhabi D., Bras R.L., Ivanov V.Y., van Horne M.P., Grassotti C., Hoffman R.N. (2006). Extending the predictability of hydrometeorological flood events using radar rainfall nowcasting. J. Hydrometeorol..

[15] Smith J.A., Baeck M.L., Meierdiercks K.L., Miller A.J., Krajewski WF. (2007). Radar rainfall estimation for flash flood forecasting in small urban watersheds. Adv. Water Resour..

[16] Villarini G., Smith J.A, Baeck M.L., Sturdevant-Rees P., Krajewski W.F. (2010). Radar analyses of extreme rainfall and flooding in urban drainage basins. J. Hydrol..

[17] Costa J.E. (1987). A comparison of the largest rainfall-runoff floods in the United States with those of the People’s Republic of China and the world. J. Hydrol..

[18] Perry C.A. (2000). Significant Floods in the United States during the 20th Century: USGS Measures a Century of Floods; USGS Fact Sheet FS-024-00.

[19] O’Connor J.E., Costa J.E. (2003). Large Floods in the United States: Where They Happen and Why.

[20] Gaume E. (2006). Post Flash-Flood Investigation-Methodological Note; Report D23Ð2.

[21] Jarrett R.D., Costa J.E. (2006). 1976 Big Thompson Flood, Colorado-Thirty Years Later; U.S. Geological Survey Fact Sheet 2006–3095.

[22] Costa J.E., Jarrett R.D. (2008). An Evaluation of Selected Extraordinary Floods in the United States Reported by the U.S. Geological Survey and Implications for Future Advancement of Flood Science.

[23] Gaume E., Borga M. (2008). Post-flood field investigations in upland catchments after major flash floods: Proposal of a methodology and illustrations. J. Flood Risk Manag..

[24] Gaume E., Bain V., Bernardara P., Newinger O., Barbuc M., Bateman A., Blaskovicova L., Bloschl G., Borga M., Dumitrescu A. (2009). A collation of data on European flash floods. J. Hydrol..

[25] Marchi L., Borga M., Preciso E., Sangati M., Gaume E., Bain V., Delrieu G., Bonnifait L., Pogačnik N. (2009). Comprehensive post-event survey of a flash flood in Western Slovenia: Observation strategy and lessons learned. Hydrol. Process..

[26] Marchi L., Borga M., Preciso E., Gaume E. (2010). Characterisation of selected extreme flash floods in Europe and implications for flood risk management. J. Hydrol..

[27] Borga M., Boscolo P., Zanon F., Sangati M. (2007). Hydrometeorological analysis of the August 29, 2003 flash flood in the Eastern Italian Alps. J. Hydrometeorol..

[28] Norbiato D., Borga M., Degli Esposti S., Gaume E., Anquetin S. (2008). Flash flood warning based on rainfall depth-duration thresholds and soil moisture conditions: An assessment for gauged and ungauged basins. J. Hydrol..

[29] Norbiato D., Borga M., Dinale R. (2009). Flash ﬂood warning in ungauged basins by use of the ﬂash ﬂood guidance and model-based runoff thresholds. Meteorol. Appl..

[30] Rossa A., Laudanna Del Guerra F., Borga M., Zanon F., Settin T, Leuenberger D. (2010). Radar-driven high-resolution hydro-meteorological forecasts of the 26 September 2007 Venice flash flood. J. Hydrol..

[31] Braud I., Roux H., Anquetin S., Maubourguet M.M., Manus C., Viallet P., Dartus D. (2010). The use of distributed hydrological models for the Gard 2002 flash flood event: Analysis of associated hydrological processes. J. Hydrol..

[32] Fread D.L. National Weather Service River Mechanics: Some Recent Development. Proceedings of the U.S./PRC Flood Forecasting Symposium/Workshop.

[33] Carpenter T.M., Georgakakos K.P. (1993). GIS-Based Procedures in Support of Flash Flood Guidance; Iowa Institute of Hydraulic Research Report No. 366.

[34] Carpenter T.M., Sperfslage J.A., Georgakakos K.P., Sweeney T., Fread D.L. (1999). National threshold runoff estimation utilizing GIS in support of operational flash flood warning systems. J. Hydrol..

[35] Hall M.J., Zaki A.F., Shahin M.A. (2001). Regional analysis using the geomorphoclimatic instantaneous unit hydrograph. Hydrol. Earth Syst. Sci..

[36] Kyiamah G.K. (1996). Monitoring and Characterization of Flash Floods. M.S. Thesis.

[37] Bhaskar N.R., French B.M., Kyiamah G.K. (2000). Characterization of flash floods in Eastern Kentucky. J. Hydrol. Eng. ASCE.

[38] Jung J.C. (2000). The Study on Estimation of the Flash Flood Index for the Bo-Chun River Basin. M.S. Thesis.

[39] Kim E.S., Choi H.I. (2011). Assessment of vulnerability to extreme flash floods in design storms. Int. J. Environ. Res. Public Health.

[40] Wonju City Government (2007). The Basic Plan Report for the Oui-mi River Maintenance Works.

[41] Chungcheongnam Province Government (2004). The Basic Plan Report for the Mae-gok River Maintenance Works.

[42] U.S. Army Corps of Engineers (USACE) (2000). Hydrograph Modeling System; Technical Reference Manual.

[43] U.S. Natural Resources Conservation Service (NRCS) (1986). Urban Hydrology for Small Watersheds; Technical Release 55 (TR-55).

[44] Clark C.O. (1945). Storage and the unit hydrograph. Trans. ASCE.

[45] Williams G.P. (1978). Bankfull discharge of rivers. Water Resour. Res..

[46] Wolman M.G., Leopold L.B. (1957). River Flood Plains: Some Observations on Their Formation; Professional Paper 282-C.

